# Reactive Hypoglycemia in Patients Prior to Cataract Surgery: A Case Series

**DOI:** 10.7759/cureus.55126

**Published:** 2024-02-28

**Authors:** Suwarna Suman, Arushi Kumar, Manoj Kumar, Indu Saxena

**Affiliations:** 1 Ophthalmology, All India Institute of Medical Sciences, Jodhpur, Jodhpur, IND; 2 Otolaryngology, Nalanda Medical College Patna, Patna, IND; 3 Physiology, Maharshi Vashishtha Autonomous State Medical College, Basti, Rampur, IND; 4 Biochemistry, All India Institute of Medical Sciences, Gorakhpur, Gorakhpur, IND

**Keywords:** preoperative anxiety, oral glucose tolerance test, postprandial hypoglycemia, late-reactive hypoglycemia, idiopathic hypoglycemia, cataract surgery

## Abstract

Cataract surgery is one of the most frequently performed surgical procedures and is often performed under topical anesthesia in conscious patients. Sweating, palpitations, and anxiety may be seen in patients about to undergo surgery. However, these are typical adrenergic symptoms of hypoglycemia and should be further investigated if occurring before surgery. Here, we report five cases of postprandial or reactive hypoglycemia observed in hospital settings just before conducting cataract surgeries in non-diabetic 52-78-year-old patients from 2019 to 2023.

## Introduction

Most cases of cataract surgeries are performed under local anesthesia on conscious patients in non-fasting conditions [1]. It is common to witness preoperative anxiety and slight tachycardia in patients about to undergo surgery. Many of these symptoms are also seen in hypoglycemia [2]. It is better to resolve hypoglycemia before the surgery for the patient’s comfort.

Hypoglycemia can be clinically defined according to the Whipple triad: low blood glucose level (<55 mg/dL), often associated with adrenergic symptoms (diaphoresis, tremors, anxiety/irritation, palpitations, dizziness, nausea, tingling, hunger), and/or neuroglycopenic symptoms (difficulty in concentration/confusion, headache, irrational behavior/anger/moodiness, vision changes, dizziness, slurred speech, loss of consciousness, pupillary sluggishness, seizures, decreased response to noxious stimuli). Resolution of symptoms should occur with an increase in plasma glucose level [2]. Asymptomatic hypoglycemia may be seen in patients with type 1 diabetes mellitus and neonates [3,4]. Based on blood glucose levels, hypoglycemia may be classified into mild (level 1, blood glucose 55-70 mg/dL), moderate (level 2, blood glucose <54 mg/dL), or severe (level 3, blood glucose <40 mg/dL) [5]. Hypoglycemia can be classified into fasting and reactive (postprandial) based on the duration between the last meal and the appearance of symptoms, as well as associated conditions [6].

Here, we describe five cases scheduled for cataract surgery who displayed symptoms of hypoglycemia just before the surgery.

## Case presentation

Table 1 summarizes the clinical findings of the cases. Three patients had normal body mass index (BMI) while two patients had a BMI of >30 kg/m^2^. All patients were non-diabetic, non-alcoholic, were not taking any regular medicines, and did not have a history of gut surgery. Symptoms of hypoglycemia occurred 2-2.5 hours after breakfast, except in the case of patient 3, a 66-year-old male, who had eaten breakfast about 4.5 hours before reporting the symptoms. Symptoms of hypoglycemia appeared when patients were being prepared for surgery. Patient 2, a 78-year-old female, complained of nausea just before the operation. A 66-year-old male requested postponement of the operation as he did not feel “too good.” The other patients did not mention anything but sweating, rapid breathing, and tremors in fingers were noticed before the operation.

**Table 1:** A summary of patient details at the time of first visit to the outpatient department.

Case	Place	Year	Age (years)	Gender	Weight (kg)	Pulse rate (per minute)	Blood pressure (mmHg)	Condition	Random blood glucose (mg/dL)
1	Jodhpur	2019	62	Female	55	72	124/82	Bilateral cataract	96
2	Jodhpur	2021	78	Female	76	68	140/88	Bilateral cataract	142
3	Jodhpur	2021	66	Male	96	72	142/84	Right eye cataract	126
4	Jodhpur	2023	52	Female	63	74	130/86	Left eye cataract	94
5	Jodhpur	2023	58	Male	67	70	140/86	Bilateral cataract	100

In all cases, pulse and blood pressure were recorded and blood samples were sent to the lab for testing. Patients were requested to rest on a hospital couch and were given sweet biscuits with tea or approximately 40 g of oral glucose in water. Three patients (patients 2, 3, and 5) reported they had felt such symptoms earlier but had dismissed them as they resolved spontaneously. Patients reportedly felt better after consuming snacks. During the hypoglycemic episode, an increase in heart rate and blood pressure was observed. The blood glucose analysis revealed a dip in blood glucose level during the episode and an increase after consuming sweet snacks/glucose. Table 2 summarizes the findings recorded during hypoglycemic episodes and half-hour post-recovery. Surgery was postponed in all cases.

**Table 2:** Findings during hypoglycemic episode and half-hour post-recovery. BMI: body mass index

Case	BMI (kg/m^2^)	During hypoglycemic episode			Half-hour post-recovery		
		Pulse (per minute)	Blood pressure (mmHg)	Blood glucose (mg/dL)	Pulse (per minute)	Blood pressure (mmHg)	Blood glucose (mg/dL)
1	25.4	108	128/84	52	76	128/82	83
2	32.9	102	146/92	56	84	144/88	110
3	33.2	108	140/86	58	80	140/84	119
4	24.6	100	130/84	54	78	128/84	104
5	22.6	98	142/86	63	70	142/84	98

An oral glucose tolerance test (OGTT) was recommended in all cases, which confirmed the diagnosis of hypoglycemia (Table 3). Interestingly, although the patients’ blood glucose levels decreased during OGTT, none of them developed visible symptoms of hypoglycemia. Patients 2 and 3 reported nausea, while patients 1 and 5 reported a mild headache, which resolved after a meal.

**Table 3:** Results of the oral glucose tolerance test (blood glucose values are in mg/dL).

Case number	Fasting glucose (0 time)	Blood glucose after 75 g glucose in 250 mL water (taken over 5 minutes)				
		1 hour	2 hour	3 hour	4 hour	5 hour
1	72	68	55	68	102	74
2	108	129	52	97	103	111
3	112	137	92	67	55	44
4	86	89	58	54	95	90
5	78	94	56	62	76	76

As the hypoglycemic event occurred more than four hours after a meal in patient 3, it could be due to a pre-diabetic condition. The patient refused further tests as the condition did not hamper his working. In the other patients, idiopathic reactive hypoglycemia was suspected, as the hypoglycemic event occurred about 2.5 hours after a meal. None of the patients wanted further investigations (estimation of plasma insulin, C-peptide, beta-hydroxybutyrate). They were, therefore, advised to consult a physician in case of worsening symptoms. The surgery was rescheduled, and patients were advised to have a meal with complex carbohydrates, protein, and fats and to avoid sugar. Blood glucose was estimated using a glucometer just before the surgery and was found to be normal. No intra or postoperative complications were observed in any case.

## Discussion

**Figure 1:**
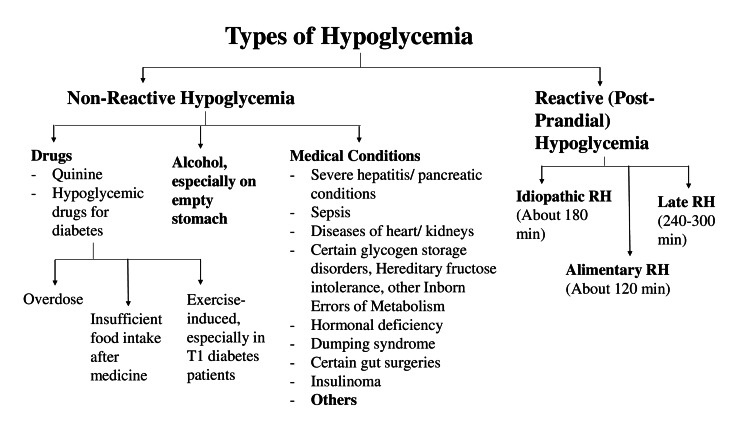
Types of hypoglycemia based on the time of occurrence after a meal. RH: reactive hypoglycemia Image credit: Manoj Kumar

Reactive or postprandial hypoglycemia [7] is a decrease in blood glucose occurring within five hours after a meal. Figure 1 shows the different types of hypoglycemia based on time of occurrence: non-reactive hypoglycemia (due to drugs, alcohol intake, or medical conditions) and reactive hypoglycemia (occurring after a meal). Reactive hypoglycemia probably occurs due to the overproduction of insulin after a meal. It is further classified into alimentary hypoglycemia (occurring within two hours after a meal), idiopathic reactive hypoglycemia (occurring within two to three hours), and late reactive hypoglycemia (occurring within three to five hours after a meal). Except for patient 3 in whom the symptoms of hypoglycemia occurred more than four hours after a meal, all patients appeared to have idiopathic hypoglycemia [8]. Idiopathic hypoglycemia is usually not an indication of future diabetes. Patient 3 had a BMI of >30 kg/m^2^ and developed symptoms more than four hours after a meal, suggesting late reactive hypoglycemia, which can be due to insulin resistance and may predict future diabetes. The prevalence of late reactive hypoglycemia is difficult to ascertain, as many patients fail to recognize or report symptoms.

Idiopathic hypoglycemia can be differentiated from idiopathic postprandial syndrome in which the symptoms are present without the decrease in blood glucose [9]. Thus, the diagnosis of hypoglycemia should be based on the presence of the Whipple triad.

Management of reactive hypoglycemia begins with recognizing the symptoms. During the episode, intake of glucose syrup or food containing fast-acting carbohydrates can relieve the symptoms. The 15-15 rule, i.e., eating 15 g of fast-acting carbohydrates and checking blood glucose after 15 minutes [5,10], can be used to treat mild hypoglycemia. If it is still below 70 mg/dL, another serving is recommended. After the resolution of symptoms, a small snack or meal is recommended to prevent blood sugar from spiking and falling again.

Symptoms of hypoglycemia were observed in more than 20 patients without a fall in blood glucose to below 70 mg/dL. These could be due to anxiety or a manifestation of the idiopathic postprandial syndrome. The clinician should, therefore, carefully note the patient’s demeanor before performing surgical procedures that do not require fasting. A thorough history of previous episodes and evaluation of the blood glucose level with a glucometer can help in diagnosis and avoid the possibility of unnecessary discomfort and stress to the patient during the surgery. A high-fiber diet reduces the intensity of hypoglycemic episodes.

## Conclusions

Reactive or postprandial hypoglycemia is low blood glucose usually two to five hours after a meal. This is different from fasting hypoglycemia and hypoglycemia due to medical conditions. A careful history and measurement of blood glucose levels in suspected patients at the time of the episode helps in diagnosis. A patient with anxiety, tremors, and diaphoresis may not be simply suffering from operation anxiety. Usually, medical intervention is not required. A high-fiber diet with complex carbohydrates can help reduce the symptoms.
